# Assessment of oral mucosal temperature as a diagnostic marker for systemic inflammation among dental patients

**DOI:** 10.6026/973206300212613

**Published:** 2025-08-31

**Authors:** Sanjaya P.R, Peyush Pratap Singh Sikarwar, Himani Painuly, Kiran Suradkar, Jayata Dhawan, Renu Dagur

**Affiliations:** 1 Department of Basic Dental & Medical Sciences, College of Dentistry, University of Hail, Hail, 55425, Kingdom of Saudi Arabia; 2 Department of Emergency Medicine, Gajra Raja Medical College, Gwalior, Madhya Pradesh, India; 3 Department of Dentistry, Shri Guru Ram Rai Institute of Medical and Health Sciences, Shri MahantIndiresh Hospital, Dehradun, Uttarakhand, India; 4 Department of Periodontology, Bharati Vidyapeeth Deemed to be University Dental College and Hospital, Navi Mumbai, Maharashtra, India; 5 Department of Pediatrics and Preventive Dentistry, MMCDSR, Mullana, Ambala, Haryana, India; 6 Department of Pediatrics and Preventive Dentistry, Kalka Dental College, Meerut, Uttar Pradesh, India

**Keywords:** Oral mucosal temperature, systemic inflammation, non-invasive biomarker, CRP, IL-6, TNf-α, infrared thermography, ROC analysis

## Abstract

Oral mucosal temperature as a non-invasive biomarker for systemic inflammation among 180 dental patients is of interest. Systemic inflammation in patients showed significantly higher mucosal temperatures (36.8 ± 0.7°C) than controls and localized inflammation groups (p < 0.001). Strong correlations were observed with serum CRP (r = 0.78), IL-6 (r = 0.72) and TNF-α (r = 0.69). ROC analysis yielded an AUC of 0.89, with 85.3% sensitivity and 82.7% specificity at a 36.2°C cutoff. Thus, we show oral thermography as a viable, non-invasive screening tool.

## Background:

Systemic inflammation has been pointed out as an important pathophysiological mechanism underlying numerous chronic diseases such as diabetes mellitus, rheumatoid arthritis, cardiovascular disease and several types of cancer [1]. Since it became evident that inflammation is a serious contributor to the development of the illness, research endeavors to develop precise, cost-effective and easy-to-access methods of measuring inflammation have grown [2]. The traditional tool of assessing systemic inflammation is serum biomarkers (C-reactive protein (CRP), interleukin-6 (IL-6) and Tumor necrosis factor-alpha (TNF-alpha)) which require invasive blood sampling and laboratory testing [3, 4]. The oral cavity is one of those peculiar anatomical locations that can serve as a portal to the whole-body inflammatory pathologies [5]. Many studies have linked systemic inflammatory burden and associated chronic illnesses with periodontal disease which is also one of the most prevalent oral inflammatory diseases [6, 7]. The oral mucosa is an extremely vascular structure in direct contact with microbial antigens and has a rapid tissue turnover, which means that it might serve as a convenient tool to access the state of systemic inflammation [8]. Recent advancements in the temperature sensing and infrared thermography technologies have opened new grounds in terms of non-invasive physiological monitoring [9]. It has also been found that inflammatory processes increase blood flow and metabolic activity as well as vascular permeability, which leads to local temperatures changes [10]. Many pathological conditions have been reported to be associated with temperature shift in the oral cavity, including malignant lesions, periodontal disease as well as pulpal inflammation [11, 12]. The possible use of oral temperature measures as a type of diagnosis has been studied in several studies. Researchers have indicated that the intraoral temperature changes during the day and is subject to various physiological and pathological factors [13]. Studies that investigate periodontal inflammation depict that subgingival temperatures in diseased areas are lower than the healthy periodontal tissues [14]. As secondary, the association between increased local temperature and the amount of inflammatory biomarkers has been identified within the studies of the inflammatory oral mucosa conditions [15].

Despite these promising findings, a substantial number of questions remain on the relationship between the systemic inflammatory status and the oral mucosal temperature. What has not been studied much is the prospect of using oral temperature data as an indicator of systemic inflammation and most studies had focused on localized patterns of oral inflammation [16]. Moreover, the requirements of standardization of measurement procedures, as well as determination of diagnostic thresholds suited to clinical use remain to be fulfilled. Potential advantages of oral mucosa temperature would be non-invasive measurement, real-time measurements, cost effectiveness and easy clinical practice use. Such strategy can be particularly helpful in dentistry, where the screening of systemic inflammatory processes can be possible when patients come to a dentist during routine visits [17]. Therefore, it is of interest to find out correlations with accepted serum levels of inflammatory markers and to evaluate the diagnostic capability of oral mucosal temperature measurements as a biomarker of systemic inflammation in dental patients.

## Materials and Methods:

## Study design:

A total of 180 participants aged 25-65 years were recruited and divided into three groups:

[1] Group 1: Healthy controls (n=60) - participants with no systemic diseases or oral inflammatory conditions

[2] Group 2: Localized oral inflammation (n=60) - participants with periodontal disease or other localized oral inflammatory conditions without systemic involvement.

[3] Group 3: Systemic inflammation (n=60) - participants with diagnosed systemic inflammatory conditions including rheumatoid arthritis, inflammatory bowel disease, or cardiovascular disease with elevated inflammatory markers.

## Inclusion and exclusion criteria:

Inclusion criteria included adults aged 25-65 years, stable medical condition and ability to provide informed consent. Exclusion criteria comprised pregnancy, fever (>37.5°C) within 48 hours, recent oral surgical procedures, active malignancy, immunosuppressive therapy and consumption of food or beverages within 30 minutes of examination.

## Equipment and materials:

Oral mucosal temperature measurements were performed using a FLIR E8-XT infrared thermal camera (FLIR Systems, Wilsonville, OR, USA) with measurement accuracy of ±2°C and thermal sensitivity of <0.06°C. The camera was calibrated according to manufacturer specifications before each measurement session. A digital infrared forehead thermometer (Braun ThermoScan 7, Kaz Europe SA, Switzerland) was used for systemic temperature measurement.

## Experimental procedures:

All measurements were conducted in a temperature-controlled examination room (22 ± 2°C) with standardized lighting conditions. Participants were seated comfortably and instructed to refrain from speaking or mouth breathing during measurements. The oral cavity was examined and mucosal surfaces were gently dried with sterile gauze. Infrared thermographic images were captured at standardized intraoral locations: buccal mucosa adjacent to the maxillary first molar, labial gingiva of maxillary central incisors and lateral border of the tongue. Three measurements were taken at each site and the mean temperature was calculated. The thermal camera was positioned at a standardized distance of 30 cm from the target area, with the operator maintaining consistent angle and positioning using a custom-designed mounting device. Peripheral blood samples (5 mL) were collected via venipuncture from the antecubital vein using standard sterile techniques. Samples were centrifuged at 3000 rpm for 10 minutes and serum was separated and stored at -80°C until analysis. Serum CRP levels were measured using high-sensitivity immunoturbidimetric assay (Beckman Coulter AU5800, Brea, CA, USA). IL-6 and TNF-α concentrations were determined using enzyme-linked immunosorbent assay (ELISA) kits (R & D Systems, Minneapolis, MN, USA) according to manufacturer protocols.

## Statistical methods:

Statistical analysis was performed using SPSS version 28.0 (IBM Corp., Armonk, NY, USA). Normality of data distribution was assessed using the Shapiro-Wilk test. Continuous variables were expressed as mean ± standard deviation for normally distributed data or median (interquartile range) for non-normally distributed data. One-way ANOVA with post-hoc Tukey's test was used for comparing means between groups. Pearson or Spearman correlation coefficients were calculated to assess relationships between variables. Receiver operating characteristic (ROC) curve analysis was performed to evaluate diagnostic accuracy. Multivariate linear regression analysis was conducted to identify independent predictors of oral mucosal temperature. Statistical significance was defined as p < 0.05.

## Results:

The study population consisted of 180 participants with mean age of 45.2 ± 12.8 years. The distribution included 98 females (54.4%) and 82 males (45.6%). There were no significant differences in age or gender distribution between groups (p > 0.05). Participants in the systemic inflammation group had higher body mass index (28.3 ± 4.2 kg/m^2^) compared to healthy controls (24.1 ± 3.1 kg/m^2^) and localized inflammation group (25.8 ± 3.8 kg/m^2^) (p < 0.001). Mean oral mucosal temperature in the systemic inflammation group (36.8 ± 0.7°C) was significantly elevated compared to healthy controls (35.2 ± 0.4°C) and localized inflammation group (35.9 ± 0.5°C) (p < 0.001). The localized inflammation group also demonstrated significantly higher temperatures than healthy controls (p < 0.01). Site-specific analysis revealed consistent patterns across all measurement locations. Buccal mucosal temperatures were 35.1 ± 0.4°C, 35.7 ± 0.5°C and 36.6 ± 0.7°C for control, localized inflammation and systemic inflammation groups, respectively (p < 0.001). Labial gingival temperatures showed similar trends: 35.2 ± 0.4°C, 35.9 ± 0.6°C and 36.8 ± 0.8°C (p < 0.001). Lateral tongue temperatures were 35.3 ± 0.5°C, 36.1 ± 0.5°C and 37.0 ± 0.7°C (p < 0.001) (Table 1, 2 - see PDF). Serum CRP levels demonstrated progressive increases across groups: healthy controls 1.2 ± 0.8 mg/L, localized inflammation 3.8 ± 2.1 mg/L and systemic inflammation 12.7 ± 8.3 mg/L (p < 0.001). IL-6 concentrations were 2.1 ± 1.2 pg/mL, 4.8 ± 2.9 pg/mL and 15.6 ± 9.8 pg/mL, respectively (p < 0.001). TNF-α levels showed similar patterns: 3.2 ± 1.8 pg/mL, 6.1 ± 3.4 pg/mL and 18.9 ± 11.2 pg/mL (p < 0.001) (Table 3 - see PDF). Strong positive correlations were observed between oral mucosal temperature and serum inflammatory markers. The correlation between mucosal temperature and CRP was r = 0.78 (p < 0.001), with IL-6 r = 0.72 (p < 0.001) and with TNF-α r = 0.69 (p < 0.001). Among the different measurement sites, lateral tongue temperature showed the strongest correlations with all inflammatory markers (CRP: r = 0.81, IL-6: r = 0.75, TNF-α: r = 0.72, all p < 0.001) (Table 4 - see PDF). ROC curve analysis demonstrated excellent diagnostic accuracy for detecting systemic inflammation using oral mucosal temperature. The area under the curve (AUC) was 0.89 (95% CI: 0.84-0.94) for discriminating between healthy controls and systemic inflammation group. The optimal cutoff temperature of 36.2°C yielded sensitivity of 85.3% (95% CI: 74.6-92.7%) and specificity of 82.7% (95% CI: 71.2-90.8%) with positive predictive value of 80.9% and negative predictive value of 86.8%. For distinguishing between localized and systemic inflammation, the AUC was 0.82 (95% CI: 0.75-0.88) with optimal cutoff of 36.4°C providing sensitivity of 78.3% and specificity of 76.7%. Multivariate linear regression analysis identified independent predictors of oral mucosal temperature. Serum CRP level was the strongest predictor (β = 0.42, p < 0.001), followed by age (β = 0.18, p < 0.01), IL-6 level (β = 0.16, p < 0.05) and gender (β = 0.12, p < 0.05). The model explained 68.4% of the variance in oral mucosal temperature (R^2^ = 0.684, p < 0.001) (Table 5 - see PDF).

## Discussion:

Demonstrating a significant diagnostic value of the proposed oral mucosal temperature measurements as a non-invasive indicator of systemic inflammation in dental patients, the current study reflects some vital insights into the future of dental-biomarker research. Having excellent diagnostic performance features, which endorse its clinical applicability, our results observe that there are significant associations between oral mucosal temperature and well-documented serum inflammatory mediators [18]. The increase oral mucosal temperatures, observed in patients with systemic inflammation, have various interrelated physiological causes. Systemic inflammatory states are characterized by increased pro-inflammatory cytokine production, which promotes vasodilation, enhanced blood flow and high activity of the metabolism in the peripheral tissues [19, 20]. Owing to constant metabolism and extensive vascularity, the oral mucosa is a sensitive marker of such systemic alterations [21]. The difference, which is clinically important and can be properly measured using modern infrared thermography, is the fact that the temperature in patients with systemic inflammation is raised by about 1.6 C compared to healthy controls. Our correlation analysis revealed especially strong exactly these relationships among oral mucosal temperature and serum TNF-a (r = 0.69), IL-6 (r = 0.72) and CRP (r = 0.78). These findings correspond with previous findings that indicated the level of significance of these inflammatory mediators to systemic inflammation processes [22, 23]. CRP is a widely recognized marker of the systemic inflammation that is largely synthesized in the liver under the influence of IL-6. A lot of clinical validation has been given to it [24]. A high correlation of our study suggests that the changes in oral mucosal temperature are affected by the same inflammatory processes that lead to a rise in CRP. The greatest associations between lateral tongue temperature measurements and the inflammatory markers were present in the site-specific analysis. This finding can be explained by activities of high metabolism of the tongue, sufficient vascularity and also direct access to the general system [25]. Additionally, the lateral tongue locality compares greatly with other oral locations in terms of the influence of the external factors such as air movement and mechanical irritation that may lead to more consistent and precise results. The analysis of the ROC curves reveals that the method of measuring of oral mucosal temperature can be used in clinical practice with good diagnostic capabilities. Your new biomarker, whose AUC of 0.89 for detecting system inflammation, is similar to those of well-established biomarkers of inflammation, in showing a high level of discriminatory power [26]. The sensitivity and specificity measures have exceeded 80 percent and cut off temperature of 36.2 °C would be a helpful point of reference in clinical analysis. Measuring the oral mucosal temperature can therefore be an effective screening tool of systemic inflammation within dental facilities as provided by these performance characteristics. The oral temperature measurement has better advantages over the conventional evaluation of inflammatory biomarkers, as it is a non-invasive method. The traditional approaches require laboratory analysis, blood and some costs and time [27]. Conversely, infrared thermography could be easily included in any typical dental check-up, results are produced immediately and there is no preparation needed by the patient. This availability might help detect systemic inflammation at an earlier stage, particularly among patients that visit dentists regularly but do not necessarily consult medical care on a routine basis. There are possible clinical applications of oral mucosal temperature measurement beyond screening. Serial measurements were ideal in obtaining real-time responses to treatment of therapeutic interventions in patients with systemic inflammatory conditions [28]. The approach can also be employed in the studies that investigate the connection between oral and systemic well-being and determine the effectiveness of the anti-inflammatory treatment.

## Conclusion:

We show that the oral mucosal surface temperature is a potential non-invasive procedure of diagnosing whether a patient has systemic inflammation or not. The clinical usefulness of the present method is reinforced by the futuristic diagnostic performance aspects and good relationships established with proximate inflammatory biomarkers. The non-invasive, rapidness of the measurements in oral temperature measurement make it an ideal alternative to the traditional test of inflammatory biomarker and it can be a part of regular dental practice.
